# PERFORMANCE OF SIX PREDICTIVE MODELS OF DEATH OF PATIENTS HOSPITALIZED FOR DECOMPENSATED CIRRHOSIS: A MULTICENTER STUDY

**DOI:** 10.1590/S0004-2803.24612024-065

**Published:** 2025-04-04

**Authors:** Ajácio Bandeira de Mello BRANDÃO, Isadora Zanotelli BOMBASSARO, Gabriela Perdomo CORAL, Jonathan SOLDERA, Carlos KUPSKI

**Affiliations:** 1 Universidade Federal de Ciências da Saúde de Porto Alegre, Faculdade de Medicina, Programa de Pós-Graduação em Medicina: Hepatologia, Porto Alegre, RS, Brasil.; 2 Unidade de Gastroenterologia e Hepatologia, Irmandade da Santa Casa de Porto Alegre, Porto Alegre, RS, Brasil.; 3 Universidade Federal de Ciências da Saúde de Porto Alegre, Faculdade de Medicina, Programa de Pós-Graduação em Patologia, Porto Alegre, RS, Brasil.; 4 Tutor, Acute Medicine and Gastroenterology, University of South Wales, Cardiff, United Kingdom.; 5 Pontifícia Universidade Católica do Rio Grande do Sul, Faculdade de Medicina, Porto Alegre, RS, Brasil.

**Keywords:** Liver cirrhosis, mortality, end stage liver disease, clinical decision rules, Cirrose hepática, mortalidade, doença hepática terminal, regras de decisão clínica

## Abstract

**Background:**

The natural history of cirrhosis is characterized by an asymptomatic phase (compensated cirrhosis) followed by a rapidly progressive phase (decompensated cirrhosis). The ability to predict the survival of patients with cirrhosis is crucial for decision-making, some as complex as the indication for a liver transplant. Several models have been developed and validated.

**Objective:**

To analyze and compare the performance of models in predicting 90-day mortality among patients hospitalized with decompensated cirrhosis.

**Methods:**

A sample of 481 hospitalized patients, with a mean age of 59.04 years 73% male, diagnosed with decompensated cirrhosis and a mean Child-Pugh score of 9. The prognostic models were calculated based on tests performed on admission: MELD-Na, MELD-Plus, MELD 3.0, ReMELD, Refit MELD, and Refit MELD-Na. The accuracy of the models was assessed by calculating the area under the receiver operating characteristic (AUROC) curve, and their respective 95% confidence intervals. Comparisons between the areas were conducted using the DeLong test. A comparison was conducted among all scores, with a primary focus on MELD 3.0 and MELD-Plus. These specific scores were the focal points of interest.

**Results:**

The scores presented AUROC curve values of 0.703-0.758, indicating a moderate capacity to discriminate between survivors and deceased patients during the considered period. The comparison between the models did not unequivocally establish the superiority of one model over the other.

**Conclusion:**

The scores have a limited predictive ability for death within 90 days in patients with decompensated cirrhosis. Our study is unable to establish the prognostic superiority of a specific scoring system.

## HIGHLIGHTS

• This retrospective, multicenter study evaluated the accuracy of six predictive models of death within 90 days in 461 patients hospitalized for decompensated cirrhosis.

• The scores presented an area under the receiver operating characteristic curve of 0.703-0.758, indicating a good ability to discriminate between survivors and deceased patients during the considered period.

• The comparison between the models did not unequivocally establish the superiority of one model over the other.

## RESUMO

**Contexto:**

A história natural da cirrose é caracterizada por uma fase assintomática (cirrose compensada), seguida por uma fase rapidamente progressiva (cirrose descompensada). A capacidade de prever a sobrevida de pacientes com cirrose é crucial para a tomada de decisões, algumas tão complexas quanto a indicação para transplante hepático. Vários modelos foram desenvolvidos e validados.

**Objetivo:**

Analisar e comparar o desempenho de modelos na predição da mortalidade em 90 dias entre pacientes hospitalizados com cirrose descompensada.

**Métodos:**

Amostra de 481 pacientes hospitalizados, com média de idade de 59,04 anos, 73% do sexo masculino, diagnosticados com cirrose descompensada e uma média de pontuação Child-Pugh de 9. Os modelos prognósticos foram calculados com base em exames realizados na admissão: MELD-Na, MELD-Plus, MELD 3.0, ReMELD, Refit MELD e Refit MELD-Na. A acurácia dos modelos foi avaliada pelo cálculo da área sob a curva de operadores recebedores de características (AUROC), com seus respectivos intervalos de confiança de 95%. As comparações entre as áreas foram realizadas por meio do teste de DeLong. Foi realizada uma comparação entre todos os escores, com foco principal no MELD 3.0 e MELD-Plus. Estes escores específicos foram os principais pontos de interesse.

**Resultados:**

Os escores apresentaram valores de AUROC entre 0,703 e 0,758, indicando uma capacidade moderada de discriminar entre sobreviventes e pacientes falecidos no período analisado. A comparação entre os modelos não estabeleceu, de forma inequívoca, a superioridade de um modelo sobre os demais.

**Conclusão:**

Os escores apresentam capacidade preditiva limitada para morte em 90 dias em pacientes com cirrose descompensada. Nosso estudo não foi capaz de estabelecer a superioridade prognóstica de um escore específico.

## INTRODUCTION

Cirrhosis is a significant global public health issue that demands attention. It is a leading cause of death worldwide - it was associated with 2.4% of global deaths in 2019. The mortality rate for cirrhosis in Brazil, for both sexes, in all age groups, in 2019, was around 10.15 per 100,000 population, accor­ding to the World Health Organization, with a major impact due to the high amount of disability-adjusted life-years (DALYs) that it generates^1^.

The natural history of cirrhosis is characterized by an asymptomatic phase (compensated cirrhosis) followed by a rapidly progressive phase (decompensated cirrhosis)^2^ ^,^ ^3^. The transition to decompensated cirrhosis, occurs at an annual rate of 4% to 12%. However, this rate may vary according to the etiology of the liver disease^4^. Patients with decompensated cirrhosis face a challenging prognosis, with a median survival of approximately 2 years. Furthermore, these individuals have a significantly higher risk of mortality, around four times greater, than patients with compensated cirrhosis^5^ ^,^ ^6^.

Risk stratification and prognosis assessment can be particularly challenging in patients with cirrhosis due to the complex nature of the disease, which involves both hepatic and extrahepatic damage and injuries^7^. However, the ability to predict the survival of patients with cirrhosis is crucial for decision-making, some as complex as the indication for a liver transplant (LT). Several models have been developed and validated. The Model for End-Stage Liver Disease (MELD) was developed to predict the 90-day death of patients with cirrhosis undergoing transjugular intrahepatic portosystemic shunt (TIPS) and uses three variables in its calculation: total bilirubin, serum creatinine, and international normalized ratio (INR)^8^. Its predictive accuracy was also evidenced in patients with cirrhosis in general^9^ ^,^ ^10^. A refinement of the MELD was the inclusion of serum sodium in the formula^11^ ^,^ ^12^. Other MELD score modifications have been proposed, including the Revised Model for End-Stage Liver Disease (REFIT) and the Revised Model for End-Stage Liver Disease with sodium incorporation (REFIT Na)^13^, MELD-Plus^14^, MELD 3.0^15^ Revised Model for End-Stage Liver Disease (ReMELD) and Revised Model for End-Stage Liver Disease with sodium incorporation (ReMELD-Na) for the European population^16^. Except for MELD-Plus score^14^, the other scores were evaluated in transplant candidates.

The objective of this study was to analyze and compare the performance of different models as predictors of death within 90 days of patients hospitalized for decompensated cirrhosis in a cohort of patients who were not included in the development of these models.

## METHODS

### Study design and population

This historical cohort study was conducted in three university hospitals located in Southern Brazil: Santa Casa de Porto Alegre, Hospital Geral de Caxias do Sul, and Hospital São Lucas da Pontifícia Universidade Católica do Rio Grande do Sul. The study included a convenience sample of patients over 18 years old hospitalized for decompensated cirrhosis. Patients hospitalized for elective procedures or admissions unrelated to complications of cirrhosis, those with hospitalization of less than 72 hours, with insufficient data to calculate the scores or follow-up of less than 90 days, were excluded from the study. For patients who experienced multiple hospitalizations during the study period, data from their initial hospitalization were utilized. The data collection period spanned from January 1, 2012, to September 30, 2020. The rates of overall survival were analyzed on December 31, 2020. 

Acute cirrhotic decompensation was defined as the occurrence of spontaneous bacterial peritonitis (SBP), variceal bleeding, grade 2 or grade 3 HE, ascites, with or without concurrent complications, or any combination of these conditions.

Diagnosis of cirrhosis was established by liver biopsy or liver elastography. However, in most cases, the diagnosis relied on a combination of clinical manifestations such as ascites, and HE, along with laboratory findings such as elevated bilirubin levels, low platelet count, prolonged prothrombin time, and decreased albumin levels. Additionally, imaging tests such as total abdominal ultrasound, computed tomography, and magnetic resonance imaging were used to support the diagnosis.

### Patient management

The investigation and treatment of patients admitted for decompensated cirrhosis followed the guidelines and recommendations set forth by hepatology societies^3^ ^,^ ^17^ ^-^ ^20^. Briefly, upon hospital admission, patients underwent an evaluation to identify any potential sources of infection, performing laboratory tests, obtaining cultures, and performing imaging studies. If an infectious focus was identified, appropriate treatment was administered, taking into consideration the microbiological profile of the institution whenever possible. Diagnostic paracentesis was conducted on all patients with ascites upon admission, or whenever clinical suspicion of SBP arose. The diagnosis of SBP was confirmed when the ascitic fluid analysis revealed a neutrophil count greater than 250 neutrophils/mm^3^, in the absence of an intra-abdominal source of infection, even if the culture yielded negative results. Patients diagnosed with SBP upon admission received treatment with a third-generation cephalosporin, in addition to intravenous albumin (1.5 g/kg on day 1 and 1.0 g/ kg on day 3)^21^ ^,^ ^22^. For patients experiencing active variceal bleeding, the treatment protocol involved terlipressin administration, along with antibiotics (ceftriaxone or norfloxacin), and immediate emergency endoscopy once their condition stabilized. Esophageal varices were treated with endoscopic banding whenever possible, and the remaining cases with sclerotherapy^23^ ^,^ ^24^. The severity of HE was assessed and classified based on the West-Haven criteria. Precipitating factors were thoroughly investigated, and if identified, appropriate treatment and/or elimination measures were implemented. In parallel, lactulose was prescribed, and its dosage was adjusted as necessary. Hospitalized patients with ascites received treatment involving a moderately sodium-restricted diet (approximately 4.6-6.9 g of salt) along with diuretics (spironolactone/furosemide). The dosages of diuretics were adjusted to achieve a daily reduction of 500 mg in patients without lower limb edema, and 1 kg per day in patients presenting with lower limb edema. Patients diagnosed with stage 2 or 3 HRS with AKI (HRS-AKI) received treatment comprising albumin and terlipressin. Terlipressin was administered at a dosage of 0.5-1.0 mg every 4-6 hours, with a gradual increase based on the patient’s response (up to a maximum dose of 12 g/day) for a duration of up to 14 days^25^ ^,^ ^26^.

### Data collection

The following variables were analyzed: demographic information (age and sex), the etiology of the underlying liver disease (hepatitis B virus, hepatitis C virus, alcohol-related liver disease, hemochromatosis, primary biliary cholangitis, primary sclerosing cholangitis, autoimmune hepatitis, MASLD, cryptogenic, other), reason for hospitalization (ascites, SBP, HE, HRS, variceal bleeding, infections other than SBP, hepatocellular carcinoma [HCC], alcoholic hepatitis), comorbidities (arterial hypertension, type 2 diabetes mellitus, chronic kidney disease), cause of death (infection, variceal bleeding, HRS, HCC, cardiovascular disease, other or unknown), and laboratory evaluation on admission (creatinine, total bilirubin, sodium, INR, albumin, blood count).

### Prognostic models

The following prognostic models were calculated based on tests performed on admission: MELD-Na^27^, MELD-Plus^14^ MELD 3.0^15^, ReMELD^16^, Refit MELD, and Refit MELD-Na^13^. For reference, the formulas used in these prognostic models are provided in APPENDIX 1.

### Primary outcome

The primary outcome of this study was defined as mortality within 90 days following the admission of patients with decompensated cirrhosis.

### Statistical analysis

Baseline patient characteristics were described using standard statistical methods. Continuous variables were compared using either the Student’s *t*-test or the Mann-Whitney test in cases where distributional assumptions were uncertain. Categorical variables were evaluated using the chi-square test or Fisher’s exact test, as applicable. 

The accuracy of the prognostic models was assessed by calculating the area under the receiver operating characteristic (AUROC) curve, accompanied by their respective 95% confidence intervals (CI). Comparisons between the areas were conducted using the DeLong test, without adjustments for multiple comparisons. A comparison was conducted among all scores, with a primary focus on MELD 3.0 and MELD-Plus. The DeLong test, a non-parametric approach, is used to compare the areas under two correlated ROC curves, providing a statistical method to evaluate whether the difference in performance between two models is significant across the entire range of thresholds. This test accounts for variability in the curves, making it particularly suitable for assessing differences in model accuracy. 

These specific scores were the focal points of interest, highlighting statistically significant differences through the presented comparisons. The analyses were performed using IBM-SPSS version 25.0 (SPSS Inc., Chicago, IL, USA), MedCalc Software version 22.01, and R program version 4.0. Statistical significance was defined as *P*-values <0.05. 

### Ethical considerations

This study adhered to the guidelines for reporting observational studies outlined by the Strengthening the Reporting of Observational Studies in Epidemiology (STROBE) initiative^28^.

The study protocol was approved by the Ethics Committee of the Santa Casa de Porto Alegre (approval number: 4,967,942). Given the observational nature of the study and retrospective data collection, an Informed Consent Form was deemed unnecessary. To ensure the ethical considerations and confidentiality of the data, the researchers signed a Confidentiality Term.

## RESULTS

Between January 1, 2012, and September 30, 2020, a total of 481 patients were hospitalized due to decompensated cirrhosis and were included in this study. Table 1 provides an overview of the demographic and clinical characteristics of the cohort, with a distinction made between survivors and individuals who died within 90 days of hospitalization. Most patients were male and had an average age of 59 years. The leading causes of cirrhosis were alcohol, which was notably more prevalent among those who did not survive, and hepatitis C virus (HCV). The primary reason for hospitalization was digestive hemorrhage attributed to varices secondary to portal hypertension, while fatalities were significantly more frequent among patients hospitalized for HE and HRS. Compared to patients who survived, patients who died within 90 days presented, upon admission, significantly higher values of serum creatinine, total bilirubin, serum sodium, INR, leukocytes, neutrophils, and monocytes. Additionally, a greater frequency of fatalities was observed among hospitalized patients with lower albumin levels.

**TABLE 1:** Characteristic of the study population (n=481).

Characteristic	Population (n=481)	Alive (n=300)	Deceased (n=181)	***P* value**
Age on admission (years)	59.04±11.5	58.7±11.1	59.9±11.6	0.257
Male	354 (73.5)	222 (74.0)	132 (72.9)	0.796
Etiology of liver disease				
Hepatitis B-	16 (3.3)	5 (1.7)	11 (6.1)	0.015
Hepatitis C	202 (42.4)	135 (45.0)	67 (37.0)	0.087
Alcohol	273 (57.3)	152 (50.7)	121 (66.9)	<0.001
MASH	12 (2.5)	8 (2.7)	4 (2.2)	>0.999
Other	56 (11.6)	43 (14.3)	13 (7.2)	0.019
Admission diagnosis type,				
Ascites	115 (24.0)	71 (23.7)	44 (24.3)	0.912
Variceal bleeding	170 (35.3)	118 (39.3)	52 (28.7)	0.019
SBP	57 (11.9)	30 (10.0)	27 (14.9)	0.111
HE	101 (21.0)	53 (17.7)	48 (26.5)	0.028
HRS	33 (6.9)	11 (3.7)	22 (12.2)	<0.001
Infections other than SBP	91 (19.0)	60 (20.0)	31 (17.1)	0.472
Comorbidities				
Arterial hypertension	130 (27.0)	82 (27.3)	48 (26.5)	0.916
Diabetes mellitus	111 (23.1)	71 (23.7)	40 (22.1)	0.738
Chronic kidney disease (renal failure)	13 (2.7)	5 (1.7)	8 (4.4)	0.085
Laboratory values				
Creatinine (mg/dL)	n=472	n=294	n=178	
	1.10	0.98	1.53	<0.001
	(0.80-1.80)	(0.77-1.40)	(1.00-2.70)	
Total bilirubin (mg/dL)	n=467	n=292	n=175	
	2.40 (1.10-4.70)	1.80 (1.00-3.50)	3.20 (1.50-7.70)	<0.001
Sodium (mEq/L)	n=472	n=296	n=176	
	137 (133-140)	138 (134-141)	136 (132-140)	0.001
INR	n=472	n=295	n=177	
	1.47 (1.27-1.70)	1.40 (1.23-1.60)	1.57 (1.31-1.91)	<0.001
Albumin (g/dL)	n=440	n=277	n=163	
	2.70 (2.33-3.20)	2.80 (2.50-3.20)	2.52 (2.20-3.00)	<0.001
Leucocytes/mm^3^	n=477	n=298	n=179	
	7220 (5155-10760)	6755 (4608-9618)	8200 (5990-12350)	<0.001
Lymphocytes/mm^3^	n=440	n=295	n=175	
	1041 (706-1597)	1039 (706-1599)	1060 (719-1590)	0.728
Neutrophils/mm^3^	n=469	n=294	n=175	
	587 (351-927)	4495 (2783-7133)	5771 (4012-9787)	<0.001
Monocytes/mm^3^	n=466	n=291	n=175	
	587 (351-827)	537 (328-844)	760 (412-1049)	0.001

Data are presented as mean ±standard deviation, median (interquartile range: p25 - p75) and counts (percentages). Mash: metabolic dysfunction-associated steatohepatitis; sbp: spontaneous bacterial peritonitis; he: hepatic encephalopathy; hrs: hepatorenal syndrome; inr: international normalized ratio.

Infection was the most common cause of death, followed by variceal bleeding secondary to portal hypertension (Table 2).

**TABLE 2:** Cause of death in 181 patients.

Cause of death	n (%)
Infection	55 (30.4)
Variceal bleeding	36 (19.9)
HRS	25 (13.8)
HCC	23 (12.7)
Cardiopulmonary disease	16 (8,8)
Unknown	26 (14.4)

HRS: hepatorenal syndrome; HCC: hepatocellular carcinoma.

**FIGURE 1:**
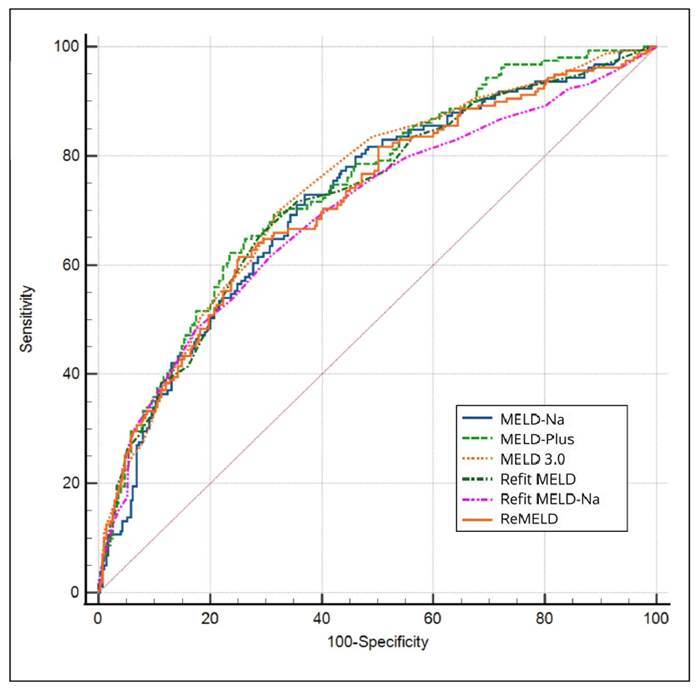
AUROC of MELD-Na, MELD-Plus, MELD 3.0, Refit MELD (Revised Model for End-Stage Liver Disease), Refit MELD-Na (Revised Model for End-Stage Liver Disease incorporating sodium), and ReMELD (Revised Model for End-Stage Liver Disease for the European population) in predicting 90-day mortality in patients hospitalized with decompensated cirrhosis.

Table 3 presents an in-depth analysis of the computed scores, along with their respective AUROC values, 95% CI, and p-values in relation to MELD-Plus and MELD 3.0, and the optimal cut-off points for predicting 90-day mortality for each model. This analysis revealed that MELD-Plus yielded an AUROC of 0.758, and notably, it exhibited no significant differences when compared to MELD 3.0, with an AUROC of 0.737. In contrast, MELD-Na, encompasses four variables and demonstrated an AUROC of 0.728. Significantly MELD 3.0 (*P*<0.01) was statistically superior compared with MELD-Na (*P*<0.01) with an AUROC of 0.737. Regarding ReMELD, it presented an AUROC of 0.731, and its performance was comparable to MELD-Plus (no significant difference) but differed from MELD 3.0 (*P*=0.05). Similarly, ReFit MELD demonstrated an AUROC of 0.734 and did not significantly differ from either MELD-Plus or MELD 3.0. Lastly, ReFit MELD-Na featured an AUROC of 0.703, and it did not significantly differ from either MELD-Plus or MELD 3.0. These findings provide valuable insights into the predictive performance of various scores for 90-day mortality, indicating largely similar performance across the variables, as shown on Figure 1. 

**TABLE 3:** AUROCs for different predictive models of death within 90 days after hospitalization for decompensated cirrhosis and ideal cut-off.

Model	Patients (N)	No. of model variables	AUROC (95%CI)	***P* value vs MELD-Plus**	***P* value vs MELD 3.0**	Youden associated criterion cut-off	Sensitivity	Specificity
MELD-Plus	481	7	0.758	-	0.84	>0.24	64%	78%
			(0.717-0.796)					
MELD 3.0	432	6	0.737	0.84	-	>20	69%	67%
			(0.693-0.778)					
ReFit MELD	458	3	0.734	0.30	0.12	> 8	66%	71%
			(0.691-0.774)					
ReMELD	458	3	0.731	0.17	0.05	>18	62%	75%
			(0.688-0.771)					
MELD-Na	453	4	0.728	0.24	<0.01	>18	73%	63%
			(0.684-0.768)					
ReFit MELD-Na	453	4	0.703	0.12	0.08	>15	61%	69%
			(0.659-0.745)					

AUROC: Area under the receiver operating characteristic curve; MELD: Model for End-Stage Liver Disease; ReFit MELD: Revised Model for End-Stage Liver Disease; ReMELD: Revised Model for End-Stage Liver Disease for the European population; MELD-Na: Model for End-Stage Liver Disease with serum sodium incorporation; ReFit MELD-Na: Revised Model for End-Stage Liver Disease with sodium incorporation.

## DISCUSSION

This observational multicenter study assessed the effectiveness of six distinct death predictor scores in adult patients undergoing hospitalization for decompensated liver cirrhosis, regardless of their transplant listing status^13^ ^-^ ^16^ ^,^ ^27^ ^,^ ^29^. It is worth highlighting that, except for MELD-Plus, the other scoring systems were initially devised within cohorts of patients awaiting LT. To our knowledge, this is the first time that the MELD Plus^14^ and ReMELD^16^ models have been tested in a Brazilian cohort.

The accuracy of the scores, assessed by the AUROC curve, showed that they are tools with moderate predictive ability for 90-day mortality in cirrhotic patients hospitalized for decompensated cirrhosis. Although AUC values between 0.7 and 0.8 have been labeled as poor, moderate, fair, or good, and these labels have been the subject of criticism^30^, we have chosen to retain the label ”moderate”, which is commonly used in the literature.

There is some evidence suggesting that artificial neural networks (ANN) serve as a promising instruments for assessing the short-term prognosis of patients with end-stage liver disease listed for LT^31^. Kartoun et al.^14^, using this approach, developed the MELD-Plus score with two versions, one with nine variables (creatinine, total bilirubin, INR, sodium, albumin, white blood cell count, age, total cholesterol, and length of stay) and the other with seven variables (excluding total cholesterol and length of stay) to estimate mortality within 90 days of patients hospitalized for cirrhosis. In the model incorporating nine variables, the AUROC curve was 0.78 (95%CI 0.75-0.81), while in the model featuring seven variables, the AUROC was 0.77 (95%CI 0.74-0.80). No statistically significant difference was observed between the two models. Significantly the score was shown to be superior to MELD and MELD-Na and works across all risk quintiles^14^ ^,^ ^32^. In our cohort, using the version with seven variables, the AUROC curve was 0.758 (95%CI 0.717-0.796). 

With the aim of correcting the gender disparity in access to LT, the US Organ Procurement and Transplant Network developed the MELD 3.0^15^, characterized by the following changes to its predecessor MELD-Na: 1) the addition of two variables, female sex, and albumin; 2) a lowered ceiling for serum creatinine from 4.0 mg/dL to 3.0 mg/dL; and 3) the inclusion of two interaction terms between albumin and creatinine and between bilirubin and sodium. The improvement in discrimination achieved with MELD 3.0 was relatively modest, as evidenced by a marginal increase of 0.007 in Harrell’s concordance statistic compared to MELD-Na^15^.

The original coefficients for the MELD variable were determined based on data from patients with liver cirrhosis who were undergoing TIPS procedures^8^. About 10 years later, the authors re-estimated the coefficients of the original MELD variables (bilirubin, creatinine, and INR) in patients who were on the waiting list for LT and implemented new upper and lower bounds for creatinine (0.8 and 3.0 mg/dL, respectively) and INR (1 and 3, respectively). The study further corroborated the significance of serum sodium as a predictor of mortality among patients with end-stage liver disease. Notably, it found that hyponatremia had the most pronounced impact in individuals with lower bilirubin values^13^. The final model, refined to incorporate the latest fitting of the four variables (bilirubin, creatinine, INR, and sodium), demonstrated a modest yet statistically meaningful enhancement in discrimination, as indicated by a concordance of 0.878 compared to 0.865 (*P*<0.01) in the validation dataset. Our research findings indicate that Refit MELD-Na is not superior to Refit MELD in its ability to predict 3-month mortality among patients with decompensated liver cirrhosis. In fact, Refit MELD-Na exhibited a lower predictive value compared to Refit MELD. The primary causes of liver cirrhosis in our patient cohort, namely HCV and alcohol, align with the etiologies observed in the study by Leise et al.^13^. As such, the etiologies do not appear to be a significant factor accounting for the observed differences in results. Furthermore, in another study conducted in Brazil, which assessed 90-day mortality among individuals admitted for decompensated cirrhosis (with HCV and alcohol also serving as the primary etiologies) and relied on tests conducted on the day of admission, no discernible improvement was seen in the predictive performance of Refit MELD and Refit MELD-Na when compared to MELD and MELD-Na. Also, Refit MELD-Na exhibited a lower predictive value compared to Refit MELD^33^. It is important to consider that both Brazilian studies focused on patients who were hospitalized due to decompensated cirrhosis, without a specific focus on those actively listed for LT. In agreement with our study, Korean researchers did not observe the superiority of Refit MELD-Na over Refit MELD, although both exhibited good predictability for 3-month mortality in patients hospitalized for cirrhosis and ascites^34^.

In 2021, the MELD score refitted to Eurotransplant data was published. A total of 6,684 patients in the waiting list for LT were included. Based on training data, refit parameters were capped at creatinine 0.7-2.5 mg/dL, bilirubin 0.3-27 mg/dL, INR 0.1-2.6, and sodium 120-139 mEq/L. ReMELD and reMELD-Na showed C-indices of 0.866 and 0.869, respectively, which were significantly (*P*<0.001) higher than the 0.849 of the UNOS-MELD and 0.860 of the UNOS-MELD-Na^16^. Among the six models examined in our study, ReMELD c-statistic with a value of 0.731 puts it in fourth place.

The MELD score has garnered widespread recognition as a pivotal asset in the routine clinical proceedings of hepatology. Nonetheless, it is essential to acknowledge that the MELD system, while invaluable, is not without imperfections. As a result, several modifications have been put forth, originating other scores derived from MELD, some of them evaluated in this study. Given that the present study did not conclusively establish the superiority of one scoring system over the others, it would be judicious to adopt the modification that has undergone the most extensive testing in clinical practice, namely, the MELD-Na formulation as introduced by Kim et al.^27^. 

Our study has limitations that need to be considered. This is a retrospective study with its inherent limitations. However, it is important to note that every score evaluated in this study was developed in retrospective studies. The sample consisted of a convenience sample, and it was not possible to include all patients admitted during the period across the various centers due to administrative reasons. Therefore, a selection bias may have occurred. Moreover, there are insufficient data available to accurately determine how many patients were admitted with ACLF or developed it during hospitalization, making it impossible to calculate EASL-CLIF scores in this context. 

## CONCLUSION

The result of our study suggests that the evaluated scores have a moderate predictive ability for death within 90 days in patients hospitalized for decompensated cirrhosis. Furthermore, our study is unable to establish the prognostic superiority of a specific scoring system within this context. A prospective multicenter study is highly desirable to address this issue. 

## Figures and Tables

**APPENDIX 1.**
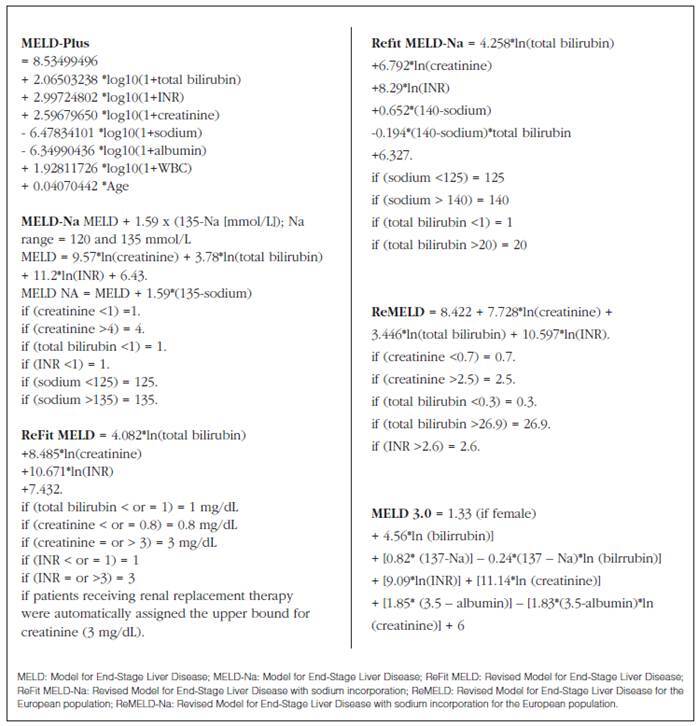
Formulas for calculating prognostic models.
